# Altitude measurement method of VHF radar based on spatial smoothing of correlation matrix

**DOI:** 10.1038/s41598-023-47294-3

**Published:** 2023-12-14

**Authors:** Xianchao Wang, Yanping Wei, Shaoyi Li, Changhui Hou, Guoming Zhang

**Affiliations:** 1 School of Information and Artificial Intelligence, Nanchang Institute of Science and Technology, Nanchang, 330108 China; 2 Nanchang Industry and Technology School, Nanchang, 330108 China

**Keywords:** Electrical and electronic engineering, Information technology

## Abstract

For very high frequency (VHF) phased array radar, the key problem to be solved in altitude measurement is the super-resolution spatial spectrum estimation under the condition of coherent sources. The spatial smoothing algorithm is a kind of decorrelation algorithm with excellent properties, but the decorrelation process is at the expense of the effective array aperture. Because it only uses the autocorrelation information of the subspace, its performance is significantly reduced, when the positions of the coherent sources are very close. In order to solve the above problems, this paper proposes an altitude measurement method of VHF radar based on the space smoothing of autocorrelation and cross-correlation matrix, which is used to realize the correlation and super-resolution processing of echo signals and multipath signals. The proposed method does not need to construct a weighting matrix, and can make full use of the received data, enhance the signal components in the equivalent spatial smoothing matrix, reduce the impact of noise, and improve the resolution of coherent sources. The simulation results show that the weighted spatial smoothing method proposed in this paper is correct and effective.

## Introduction

The very high frequency (VHF) radar has the good performance of anti stealth and anti-radiation missile (ARM) due to its long wavelength, but it also has the problems of wide beam and low angular resolution^1–3^. During the altitude measurement of targets, the pitching beam is easy to hit the ground. The received echo during the low angle tracking includes not only the direct wave signal reflected from the target, but also the multipath signal reflected from the ground (sea) surface, which will cause the lobe splitting and increase the detection and measurement error^4,5^.

The VHF phased array radar fully combines the characteristics of meter wave band and phased array system. The key problem to be solved in the low elevation altitude measurement is the super-resolution spatial spectrum estimation under the condition of coherent sources^6–9^. The spatial smoothing algorithm is a kind of the decorrelation method with excellent properties^10–12^. Through continuous improvement, it has become a common preprocessing method for processing coherent sources, but the decorrelation process is at the expense of effective array aperture^9,13^. In order to obtain larger effective array aperture, the forward and backward spatial smoothing algorithm is proposed^14^. Compared with the forward spatial smoothing algorithm, it has less array aperture loss. However, these traditional algorithms only use the autocorrelation information of subspace. When the coherent sources are close to each other, the performance of these algorithms will decrease significantly. The method proposed in this paper is to cross correlate the autocorrelation matrix output from the subarrays, and then use the covariance matrix after averaging the forward and backward cross correlation matrices as the modified spatial smoothing matrix. The proposed method is actually a weighted spatial smoothing algorithm, but it does not need to construct a weighting matrix. It can make full use of the received data, enhance the signal components in the equivalent spatial smoothing matrix, reduce the impact of noise, and improve the resolution.

The remainder of this paper is organized as follows. In Sect. “Related works”, the geometric model of spherical ground multipath and the target height measurement formula are established. Then, based on this model, the multipath echo signal model of meter wave radar altitude measurement is given. In Sect. “Methodology”, for the superresolution of coherent sources under multipath conditions, a forward and backward weighted spatial smoothing algorithm is proposed to realize the decorrelation processing of echo signals and multipath signals. In Sect. “Experiments”, the effectiveness of the proposed method is verified through simulation and comparison experiments. Finally, Sect. “Conclusion” contains a conclusion.

## Related works

In the section, the multipath geometric model of spherical ground and the multipath signal model of meter wave altimeter are presented.

### Multipath geometric model of spherical ground

**Figure 1:**
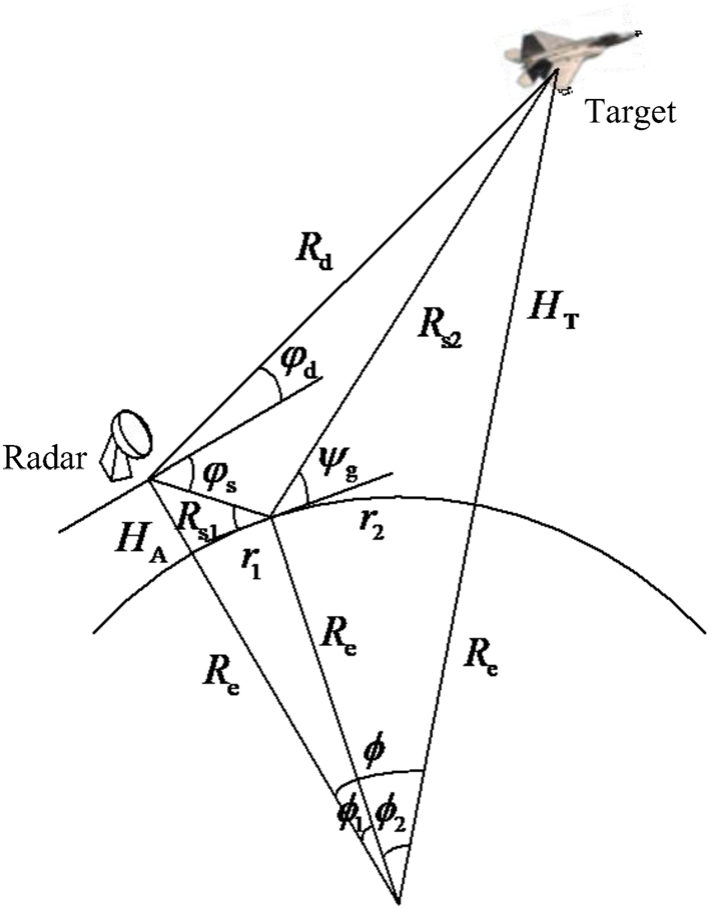
Multipath geometric model of spherical ground.

In order to obtain a more accurate signal model, especially when the target is far from the radar, it is necessary to consider the curvature of the earth itself, and also the curvature of the signal path caused by refraction in the troposphere^15,16^. The wave path curve is replaced by the effective radius of the earth $R_{{\text{e}}}$^17^, which can be expressed as

1 $$R_{{\text{e}}} = R_{0} \left( {1 + R_{0} \frac{{{\text{d}}N(h)}}{{{\text{d}}h}}} \right)^{ - 1}$$

where $R_{0}$ is the actual earth radius (6370 km), *h* represents altitude, *N*(*h*) is the atmospheric refractive index at altitude *h*, and $\frac{{{\text{d}}N(h)}}{{{\text{d}}h}}$ is the refractivity. After the selection of $R_{{\text{e}}}$, the wave path of the imaginary earth is a straight line, so it is easier to handle. Under the standard atmospheric conditions, $\frac{{{\text{d}}N(h)}}{{{\text{d}}h}} = - 4 \times 10^{ - 8} {\text{m}}^{ - 1}$. Therefore, under the standard atmospheric conditions the effective radius of the antenna is $R_{{\text{e}}} \approx \frac{4}{3}R_{0} = 8500{\text{km}}$. Figure 1 shows the geometric model of spherical ground multipath, in which $H_{{\text{A}}}$ and $H_{{\text{T}}}$ are respectively the heights of the radar and target, and $R_{d}$ is the distance between the target and radar. The arc lengths $r_{{1}}$ and $r_{{2}}$ represent the curve lengths between the projection points of the center of the array and the target on the ground and the ground reflection center point, respectively, and $\phi_{{1}}$ and $\phi_{{2}}$ represent the corresponding geocentric angles. $\phi = \phi_{{1}} + \phi_{{2}}$. $R_{{{\text{s1}}}}$ is the distance between the ground reflection center point and the center of the array, and $R_{{{\text{s2}}}}$ is the distance between the ground reflection center point and the target. $\psi_{{\text{g}}}$ represents the angle between the line connecting the target and the ground reflection center point and the horizontal plane. $\varphi_{{\text{d}}}$ and $\varphi_{{\text{s}}}$ are the incident angles of direct and reflected waves, respectively.

For the spherical ground multipath geometric model, after the elevation angle $\varphi_{{\text{d}}}$ is measured, the height of the target $H_{{\text{T}}}$ can be expressed as

2 $$H_{{\text{T}}} = \sqrt {R_{{\text{d}}}^{2} + \left( {H_{{\text{A}}} + R_{{\text{e}}} } \right)^{2} - 2R_{{\text{d}}} \left( {H_{{\text{A}}} + R_{{\text{e}}} } \right)\cos \varphi_{{\text{d}}} } - R_{{\text{e}}}$$

where $$\varphi_{{\text{d}}} = \arccos \left( {\frac{{R_{{\text{d}}}^{2} + \left( {H_{{\text{A}}} + R_{{\text{e}}} } \right)^{2} - \left( {H_{{\text{T}}} + R_{{\text{e}}} } \right)^{2} }}{{2R_{{\text{d}}} \left( {H_{{\text{A}}} + R_{{\text{e}}} } \right)}}} \right) - \frac{\pi }{2}$$.

The main lobe beam width of the antenna of the VHF radar is relatively wide, with a beam width of 10 degrees, making it easier to receive both the direct wave signals and the reflection signals simultaneously. Moreover, the direct wave signal and reflection signal are a group of coherent signals that are superimposed within the main lobe of the antenna pattern, which affects the accuracy of angle measurement. According to the formula derived above, when the target height is 10000 m, $\varphi_{{\text{d}}} \approx 2.16^{ \circ }$, and $\varphi_{{\text{s}}} \approx - 2.26^{ \circ }$. When the target height is 6000 m, $\varphi_{{\text{d}}} \approx 1.02^{ \circ }$, and $\varphi_{{\text{s}}} \approx - 1.14^{ \circ }$. From the above numerical analysis, it can be concluded that the included angle between the direct wave and the reflected wave in both cases is less than the main lobe beam width of the antenna. Therefore, the array super-resolution algorithm must be used to achieve the super-resolution of the direct wave and reflected wave.

### Multipath signal model of meter wave altimeter

**Figure 2:**
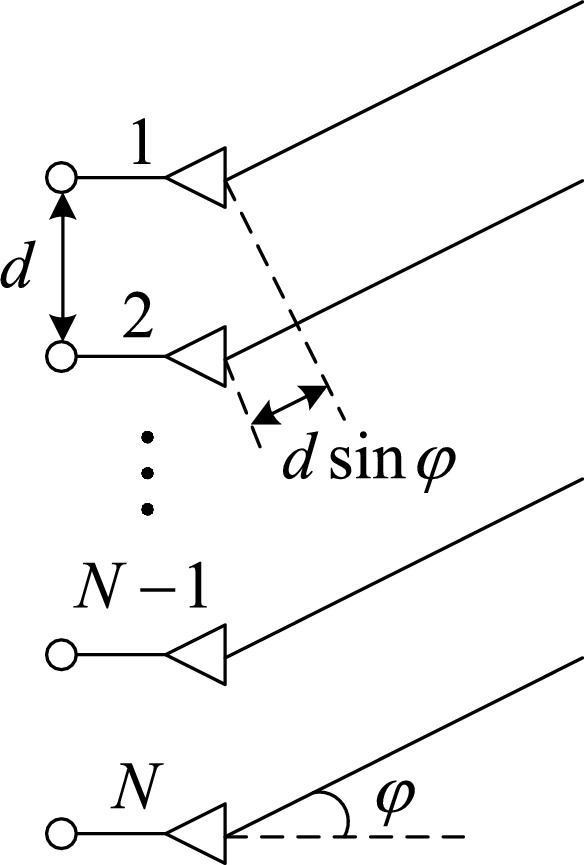
Structural model of antenna array.

Direction of arrival estimation (DOA) is to obtain DOA information of the source by using the phase difference between the source and each array element^18–20^. In the height measurement of meter wave phased array radar, only the pitch angle estimation of the source should be considered. Assume that the array structure is a uniform linear array, where the number of elements is $N$, the spacing between elements is $d$, and the corresponding array structure is shown in Fig. 2.

Suppose the narrowband point source $s_{k} (t) \, (k = 1,2, \cdots ,K)$ is incident by plane wave whose wavelength is $\lambda$, the incident direction of direct wave is $\varphi_{{{\text{d}}k}}$, and the incident direction of reflected wave is $\varphi_{{{\text{s}}k}}$. The output of the direct wave signal and the reflected wave signal of the *k*th source received by the *i*th array element at the moment *t* can be expressed as

3 $$x_{ik} (t) = s_{k} \left( t \right)\exp \left( {{\text{j}}\frac{{2\pi d\left( {i - 1} \right)\sin (\varphi_{{{\text{d}}k}} )}}{\lambda }} \right) + \rho_{k} s_{k} \left( {t - \tau } \right)\exp \left( {{\text{j}}\frac{{2\pi d\left( {i - 1} \right)\sin (\varphi_{{{\text{s}}k}} )}}{\lambda }} \right) + n_{i} \left( t \right)$$

where $\tau = \frac{\Delta R}{c}$, $\Delta R$ is the wave path difference between reflected wave and direct wave, $s_{k} \left( {t - \tau } \right) = s_{k} \left( t \right)\exp \left( { - {\text{j}}\frac{2\pi }{\lambda }\Delta R} \right)$, $\rho_{k} = \left| {\rho_{k} } \right|\exp ({\text{j}}\Delta \varphi )$ is the reflection coefficient of the ground where the *k*th target reflection area is located, $\left| {\rho_{k} } \right|$ is the amplitude of the ground reflection coefficient, and $\Delta \varphi = \pi$, ${\kern 1pt} k = 1,2, \cdots ,K$.

${\varvec{A}} = \left[ {{\varvec{a}}\left( {\varphi_{{{\text{d}}1}} } \right),{\varvec{a}}\left( {\varphi_{{{\text{s}}1}} } \right),{\varvec{a}}\left( {\varphi_{{{\text{d}}2}} } \right),{\varvec{a}}\left( {\varphi_{{{\text{s}}2}} } \right), \cdots ,{\varvec{a}}\left( {\varphi_{{{\text{d}}K}} } \right),{\varvec{a}}\left( {\varphi_{{{\text{s}}K}} } \right)} \right]$ is the manifold matrix of a $N \times 2K$ dimensional array. For a uniform linear array, the array guidance vector $\left[ {{\varvec{a}}\left( {\varphi_{{{\text{d}}k}} } \right),{\varvec{a}}\left( {\varphi_{{{\text{s}}k}} } \right)} \right]$ corresponding to the source $k$ considering multipath effects has the Vandermonde property, which can be expressed as

4 $$\left[ {{\varvec{a}}\left( {\varphi_{{{\text{d}}k}} } \right),{\varvec{a}}\left( {\varphi_{{{\text{s}}k}} } \right)} \right] = \left[ {\begin{array}{*{20}c} 1 & 1 \\ {\exp \left( {{\text{j}}\frac{2\pi }{\lambda }d\sin \left( {\varphi_{{{\text{d}}k}} } \right)} \right)} & {\exp \left( {{\text{j}}\frac{2\pi }{\lambda }d\sin \left( {\varphi_{{{\text{s}}k}} } \right)} \right)} \\ \vdots & \vdots \\ {\exp \left( {{\text{j}}\frac{2\pi }{\lambda }\left( {N - 1} \right)d\sin \left( {\varphi_{{{\text{d}}k}} } \right)} \right)} & {\exp \left( {{\text{j}}\frac{2\pi }{\lambda }\left( {N - 1} \right)d\sin \left( {\varphi_{{{\text{s}}k}} } \right)} \right)} \\ \end{array} } \right]$$

where ${\varvec{a}}(\varphi_{{{\text{d}}k}} )$ and ${\varvec{a}}(\varphi_{{{\text{s}}k}} )$ are the spatial guidance vectors of the direct wave directions $\varphi_{{{\text{d}}k}}$ and $\varphi_{{{\text{s}}k}}$ of the target $k$, respectively. Considering the multipath effect for the incident signal, ${\varvec{S}}(t) = \left[ {s_{1} (t),\rho_{1} s_{1} (t - \tau ),s_{2} (t),\rho_{2} s_{2} (t - \tau ), \cdots ,s_{K} (t),\rho_{K} s_{K} (t - \tau )} \right]^{{\text{T}}}$ is the $2K \times 1$ dimensional complex amplitude vector, where $s_{k} (t) = A_{k} e^{{{\text{j}}2\pi f_{{{\text{d}}_{k} }} (m - 1)T_{{\text{r}}} }} ,{\kern 1pt} {\kern 1pt} {\kern 1pt} {\kern 1pt} {\kern 1pt} {\kern 1pt} {\kern 1pt} m = 1,2, \cdots ,M$, and $M$ is the number of snapshots.

Because each target echo contains direct wave and ground reflected wave, the complex envelope of array received signal can be expressed as

5 $${\varvec{X}}(t) = {\varvec{A}}\left( \varphi \right){\varvec{S}}\left( t \right) + {\varvec{N}}\left( t \right)$$

where ${\varvec{X}}(t) = \left[ {x_{1} (t),x_{2} (t), \cdots ,x_{N} (t)} \right]^{{\text{T}}}$ is the $N \times 1$ dimensional snapshot data vector received by the array, and ${\varvec{N}}(t) = \left[ {n_{1} (t),n_{2} (t), \cdots ,n_{N} (t)} \right]^{{\text{T}}}$ is the $N \times 1$ dimensional array noise vector.

## Methodology

**Figure 3:**
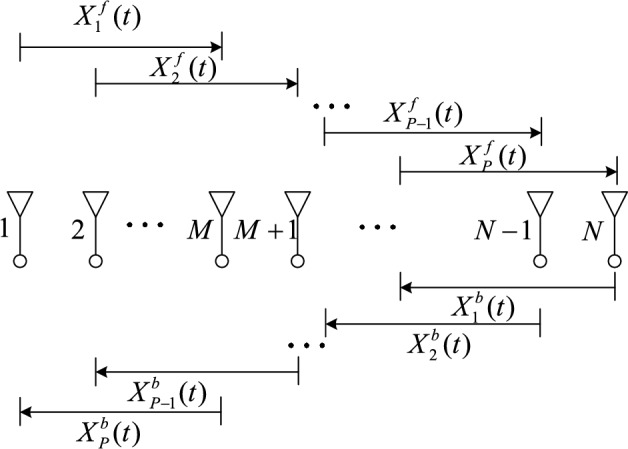
Forward and backward spatial smoothing algorithm.

Figure 3 shows the schematic diagram of the forward and backward spatial smoothing (FBSS) algorithm. The principle of the forward smoothing algorithm is as follows. Divide the $N$ uniform linear arrays into the $P$ subarrays, where the adjacent subarrays are misaligned by one element. If the number of the elements of each subarray is $M$, then the number of the subarrays is $P = N - M + 1$, and the number of the elements of the subarray is $M = N - P + 1$.

The output vector of each subarray can be expressed as follows.

6 $$\left\{ \begin{gathered} {\varvec{X}}_{1}^{f} (t) = \left[ {x_{1} (t),x_{2} (t), \cdots ,x_{M} (t)} \right]^{{\text{T}}} \hfill \\ {\varvec{X}}_{2}^{f} (t) = \left[ {x_{2} (t),x_{3} (t), \cdots ,x_{M + 1} (t)} \right]^{{\text{T}}} \hfill \\ {\kern 1pt} {\kern 1pt} {\kern 1pt} {\kern 1pt} {\kern 1pt} {\kern 1pt} {\kern 1pt} {\kern 1pt} {\kern 1pt} {\kern 1pt} {\kern 1pt} {\kern 1pt} {\kern 1pt} {\kern 1pt} {\kern 1pt} {\kern 1pt} {\kern 1pt} {\kern 1pt} {\kern 1pt} {\kern 1pt} {\kern 1pt} {\kern 1pt} {\kern 1pt} {\kern 1pt} {\kern 1pt} {\kern 1pt} {\kern 1pt} {\kern 1pt} {\kern 1pt} {\kern 1pt} {\kern 1pt} {\kern 1pt} {\kern 1pt} {\kern 1pt} {\kern 1pt} {\kern 1pt} {\kern 1pt} {\kern 1pt} {\kern 1pt} {\kern 1pt} {\kern 1pt} {\kern 1pt} {\kern 1pt} {\kern 1pt} {\kern 1pt} {\kern 1pt} {\kern 1pt} {\kern 1pt} {\kern 1pt} {\kern 1pt} {\kern 1pt} {\kern 1pt} {\kern 1pt} {\kern 1pt} {\kern 1pt} {\kern 1pt} {\kern 1pt} {\kern 1pt} {\kern 1pt} {\kern 1pt} {\kern 1pt} {\kern 1pt} {\kern 1pt} {\kern 1pt} {\kern 1pt} {\kern 1pt} {\kern 1pt} {\kern 1pt} {\kern 1pt} {\kern 1pt} {\kern 1pt} \vdots \hfill \\ {\varvec{X}}_{P - 1}^{f} (t) = \left[ {x_{P - 1} (t),x_{P} (t), \cdots ,x_{P + M - 2} (t)} \right]^{{\text{T}}} \hfill \\ {\varvec{X}}_{P}^{f} (t) = \left[ {x_{P} (t),x_{P + 1} (t), \cdots ,x_{P + M - 1} (t)} \right]^{{\text{T}}} \hfill \\ \end{gathered} \right.$$

The output vector of the $p$ th subarray can be expressed as

7 $$\begin{gathered} {\varvec{X}}_{p}^{f} (t) = \left[ {x_{p} (t),x_{p + 1} (t), \cdots ,x_{p + M - 1} (t)} \right]^{{\text{T}}} \\ { = }{\varvec{A}}_{M} {\varvec{D}}^{p - 1} {\varvec{S}}\left( t \right) + {\varvec{N}}_{p} \left( t \right) \\ \end{gathered}$$

where $p = 1,2, \cdots ,P$, and the matrix ${\varvec{D}}$ is

8 $${\varvec{D}} = \left[ {\begin{array}{*{20}c} {\exp \left( {{\text{j}}\frac{2\pi }{\lambda }d\sin \left( {\varphi_{{{\text{d1}}}} } \right)} \right)} & 0 \\ 0 & {\exp \left( {{\text{j}}\frac{2\pi }{\lambda }d\sin \left( {\varphi_{{{\text{s1}}}} } \right)} \right)} \\ \end{array} } \right]$$

Then the data covariance matrix of the $p$ th subarray is

9 $$\begin{gathered} {\varvec{R}}_{p}^{f} = E[{\varvec{X}}_{p}^{f} ({\varvec{X}}_{p}^{f} )^{H} ] \\ = {\varvec{A}}_{M} {\varvec{D}}^{p - 1} E[{\varvec{SS}}^{H} ]({\varvec{D}}^{p - 1} )^{H} {\varvec{A}}_{M}^{H} + \sigma^{2} {\varvec{I}} \\ { = }\user2{ A}_{M} {\varvec{D}}^{p - 1} {\varvec{R}}_{{\varvec{S}}} ({\varvec{D}}^{p - 1} )^{H} {\varvec{A}}_{M}^{H} + \sigma^{2} {\varvec{I}} \\ \end{gathered}$$

Therefore, the forward spatial smoothing matrix can be expressed as

10 $$\begin{gathered} {\varvec{R}}^{f} = \frac{1}{P}\sum\limits_{p = 1}^{P} {{\varvec{R}}_{p}^{f} } = {\varvec{A}}_{M} \left[ {\frac{1}{P}\sum\limits_{p = 1}^{P} {{\varvec{D}}^{p - 1} {\varvec{R}}_{{\varvec{S}}} ({\varvec{D}}^{p - 1} )^{H} } } \right]{\varvec{A}}_{M}^{H} + \sigma^{2} {\varvec{I}} \\ { = }\user2{ A}_{M} {\varvec{R}}_{{\varvec{S}}}^{f} {\varvec{A}}_{M}^{H} + \sigma^{2} {\varvec{I}} \\ \end{gathered}$$

The principle of the backward smoothing algorithm is as follows. Divide the $N$ uniform linear arrays into the $P$ subarrays, where the adjacent subarrays are misaligned by one element. If the number of elements of each subarray is $M$, then the number of the subarrays is $P = N - M + 1$, and the number of the elements of the subarray is $M = N - P + 1$. The output vector of each subarray can be expressed as follows.

11 $$\left\{ \begin{gathered} {\varvec{X}}_{1}^{b} (t) = \left[ {x_{N}^{*} (t),x_{N - 1}^{*} (t), \cdots ,x_{N - M + 1}^{*} (t)} \right]^{{\text{T}}} \hfill \\ {\varvec{X}}_{2}^{b} (t) = \left[ {x_{N - 1}^{*} (t),x_{N - 2}^{*} (t), \cdots ,x_{N - M}^{*} (t)} \right]^{{\text{T}}} \hfill \\ {\kern 1pt} {\kern 1pt} {\kern 1pt} {\kern 1pt} {\kern 1pt} {\kern 1pt} {\kern 1pt} {\kern 1pt} {\kern 1pt} {\kern 1pt} {\kern 1pt} {\kern 1pt} {\kern 1pt} {\kern 1pt} {\kern 1pt} {\kern 1pt} {\kern 1pt} {\kern 1pt} {\kern 1pt} {\kern 1pt} {\kern 1pt} {\kern 1pt} {\kern 1pt} {\kern 1pt} {\kern 1pt} {\kern 1pt} {\kern 1pt} {\kern 1pt} {\kern 1pt} {\kern 1pt} {\kern 1pt} {\kern 1pt} {\kern 1pt} {\kern 1pt} {\kern 1pt} {\kern 1pt} {\kern 1pt} {\kern 1pt} {\kern 1pt} {\kern 1pt} {\kern 1pt} {\kern 1pt} {\kern 1pt} {\kern 1pt} {\kern 1pt} {\kern 1pt} {\kern 1pt} {\kern 1pt} {\kern 1pt} {\kern 1pt} {\kern 1pt} {\kern 1pt} {\kern 1pt} {\kern 1pt} {\kern 1pt} {\kern 1pt} {\kern 1pt} {\kern 1pt} {\kern 1pt} {\kern 1pt} {\kern 1pt} {\kern 1pt} {\kern 1pt} {\kern 1pt} {\kern 1pt} {\kern 1pt} {\kern 1pt} {\kern 1pt} {\kern 1pt} {\kern 1pt} {\kern 1pt} \vdots \hfill \\ {\varvec{X}}_{P - 1}^{b} (t) = \left[ {x_{N - P + 2}^{*} (t),x_{N - P + 1}^{*} (t), \cdots ,x_{N - M - P + 1}^{*} (t)} \right]^{{\text{T}}} \hfill \\ {\varvec{X}}_{P}^{b} (t) = \left[ {x_{N - P + 1}^{*} (t),x_{N - P}^{*} (t), \cdots ,x_{N - M - P + 2}^{*} (t)} \right]^{{\text{T}}} \hfill \\ \end{gathered} \right.$$

The output vector of the backward the $p$ th subarray can be expressed as

12 $${\varvec{X}}_{p}^{b} (t) = \left[ {x_{N - p + 1}^{*} (t),x_{N - p}^{*} (t), \cdots ,x_{N - M - p + 2}^{*} (t)} \right]^{{\text{T}}}$$

The output vector of the $P - p + 1$ th backward subarray can be expressed as

13 $$\begin{gathered} {\varvec{X}}_{P - p + 1}^{b} (t) = \left[ {x_{p + M - 1}^{*} (t),x_{p + M - 2}^{*} (t), \cdots ,x_{p}^{*} (t)} \right]^{{\text{T}}} \\ { = }{\varvec{J}}_{M} \left( {{\varvec{X}}_{p}^{f} (t)} \right)^{*} \\ { = }{\varvec{J}}_{M} {\varvec{A}}_{M}^{*} {\varvec{D}}^{ - (p - 1)} {\varvec{S}}^{*} \left( t \right) + {\varvec{J}}_{M} {\varvec{N}}_{p}^{*} \left( t \right) \\ \end{gathered}$$

where $$p = 1,2, \cdots ,P$$.

Then, the data covariance matrix of the $P - p + 1$ th backward smoothing subarray is

14 $$\begin{gathered} {\varvec{R}}_{P - p + 1}^{b} = E[{\varvec{X}}_{P - p + 1}^{b} ({\varvec{X}}_{P - p + 1}^{b} )^{H} ] \\ = {\varvec{J}}_{M} {\varvec{A}}_{M}^{*} {\varvec{D}}^{ - (p - 1)} {\varvec{S}}^{*} ({\varvec{S}}^{*} )^{H} {\varvec{D}}^{p - 1} ({\varvec{A}}_{M}^{*} )^{H} {\varvec{J}}_{M} + {\varvec{J}}_{M} {\varvec{N}}_{p}^{*} ({\varvec{N}}_{p}^{*} )^{H} {\varvec{J}}_{M} \\ = {\varvec{A}}_{M} {\varvec{D}}^{ - (M - 1)} {\varvec{D}}^{ - (p - 1)} E[{\varvec{S}}^{*} ({\varvec{S}}^{*} )^{H} ]{\varvec{D}}^{p - 1} {\varvec{D}}^{M - 1} {\varvec{A}}_{M}^{H} + \sigma^{2} {\varvec{I}} \\ = {\varvec{A}}_{M} {\varvec{D}}^{ - (M + p - 2)} {\varvec{R}}_{{\varvec{S}}}^{*} {\varvec{D}}^{M + p - 2} {\varvec{A}}_{M}^{H} + \sigma^{2} {\varvec{I}} \\ \end{gathered}$$

Therefore, the backward spatial smoothing matrix can be expressed as

15 $$\begin{gathered} {\varvec{R}}^{b} = \frac{1}{P}\sum\limits_{p = 1}^{P} {{\varvec{R}}_{P - p + 1}^{b} } \\ = {\varvec{A}}_{M} \left[ {\frac{1}{P}\sum\limits_{p = 1}^{P} {{\varvec{D}}^{ - (M + p - 2)} {\varvec{R}}_{{\varvec{S}}}^{*} {\varvec{D}}^{M + p - 2} } } \right]{\varvec{A}}_{M}^{H} + \sigma^{2} {\varvec{I}} \\ = {\varvec{A}}_{M} {\varvec{R}}_{{\varvec{S}}}^{b} {\varvec{A}}_{M}^{H} + \sigma^{2} {\varvec{I}} \\ \end{gathered}$$

From the perspective of information utilization, the conventional FBSS algorithm only uses the autocorrelation information of the output of the $P$ subarrays. The more information is used, the higher the accuracy of the spatial spectrum estimation will be. The improved spatial smoothing algorithm proposed in this paper uses the cross correlation information of the output of the $P^{2}$ subarrays, and the new spatial smoothing matrix can be expressed as

16 $$\begin{gathered} {\varvec{R}}^{f4} = \frac{1}{P}\sum\limits_{p = 1}^{P} {\sum\limits_{q = 1}^{P} {{\varvec{R}}_{pq}^{f} {\varvec{R}}_{pq}^{f} } } \\ = \frac{1}{P}\sum\limits_{p = 1}^{P} {\sum\limits_{q = 1}^{P} {\left[ {{\varvec{A}}_{M} {\varvec{D}}^{p - 1} {\varvec{R}}_{{\varvec{S}}} ({\varvec{D}}^{q - 1} )^{H} {\varvec{A}}_{M}^{H} + \sigma^{2} {\varvec{I}}\delta_{pq} } \right]\left[ {{\varvec{A}}_{M} {\varvec{D}}^{p - 1} {\varvec{R}}_{{\varvec{S}}} ({\varvec{D}}^{q - 1} )^{H} {\varvec{A}}_{M}^{H} + \sigma^{2} {\varvec{I}}\delta_{pq} } \right]} } \\ = \frac{1}{P}\sum\limits_{p = 1}^{P} {\sum\limits_{q = 1}^{P} {\left[ {{\varvec{A}}_{M} {\varvec{D}}^{p - 1} {\varvec{R}}_{{\varvec{S}}} ({\varvec{D}}^{q - 1} )^{H} {\varvec{A}}_{M}^{H} {\varvec{A}}_{M} {\varvec{D}}^{p - 1} {\varvec{R}}_{{\varvec{S}}} ({\varvec{D}}^{q - 1} )^{H} {\varvec{A}}_{M}^{H} + 2\sigma^{2} {\varvec{A}}_{M} {\varvec{D}}^{p - 1} {\varvec{R}}_{{\varvec{S}}} ({\varvec{D}}^{p - 1} )^{H} {\varvec{A}}_{M}^{H} } \right]} } + \sigma^{4} {\varvec{I}} \\ = {\varvec{A}}_{M} \left\{ {\frac{1}{P}\sum\limits_{p = 1}^{P} {\sum\limits_{q = 1}^{P} {{\varvec{D}}^{p - 1} \left[ {{\varvec{R}}_{{\varvec{S}}} ({\varvec{D}}^{q - 1} )^{H} {\varvec{A}}_{M}^{H} {\varvec{A}}_{M} {\varvec{D}}^{p - 1} {\varvec{R}}_{{\varvec{S}}} } \right]({\varvec{D}}^{q - 1} )^{H} } } } \right\}{\varvec{A}}_{M}^{H} + {\varvec{A}}_{M} \left\{ {\frac{1}{P}\sum\limits_{p = 1}^{P} {{\varvec{D}}^{p - 1} (2\sigma^{2} {\varvec{R}}_{{\varvec{S}}} )({\varvec{D}}^{p - 1} )^{H} } } \right\}{\varvec{A}}_{M}^{H} + \sigma^{4} {\varvec{I}} \\ = {\varvec{A}}_{M} \left\{ {\frac{1}{P}\sum\limits_{p = 1}^{P} {\sum\limits_{q = 1}^{P} {B_{qp} {\varvec{D}}^{p - 1} {\varvec{R}}_{{\varvec{S}}} ({\varvec{D}}^{q - 1} )^{H} } } } \right\}{\varvec{A}}_{M}^{H} + {\varvec{A}}_{M} \left\{ {\frac{1}{P}\sum\limits_{p = 1}^{P} {{\varvec{D}}^{p - 1} (2\sigma^{2} {\varvec{R}}_{{\varvec{S}}} )({\varvec{D}}^{p - 1} )^{H} } } \right\}{\varvec{A}}_{M}^{H} + \sigma^{4} {\varvec{I}} \\ \end{gathered}$$

where $B_{qp} = {\varvec{S}}^{H} ({\varvec{D}}^{q - 1} )^{H} {\varvec{A}}_{M}^{H} {\varvec{A}}_{M} {\varvec{D}}^{p - 1} {\varvec{S}}$ is a real number.

The improved algorithm is to cross correlate the autocorrelation matrix output from the subarrays, and then use the covariance matrix after averaging the forward and backward cross correlation matrices as the modified spatial smoothing matrix. It is actually a weighted spatial smoothing algorithm, but it does not need to construct a weighting matrix. It can make full use of the received data, enhance the signal components in the equivalent spatial smoothing matrix, reduce the impact of noise, and improve the resolution.

The specific steps of the weighted virtual space smoothing algorithm are as follows.

Step 1. Use the data ${\varvec{X}}(t) = \left[ {x_{1} (t),x_{2} (t), \cdots ,x_{N} (t)} \right]^{{\text{T}}}$ received by the array and its conjugate information ${\varvec{X}}^{ * } (t) = \left[ {x_{1}^{ * } (t),x_{2}^{ * } (t), \cdots ,x_{N}^{ * } (t)} \right]^{{\text{T}}}$, then the $P$ forward virtual subarrays are constructed, whose output vectors can be expressed as ${\varvec{X}}_{p}^{f} (t) = \left[ {x_{p} (t),x_{p + 1} (t), \cdots ,x_{p + M - 1} (t)} \right]^{{\text{T}}}$, $p = 1,2, \cdots ,P$, and the $P$ backward virtual subarrays are constructed, whose output vectors can be expressed as ${\varvec{X}}_{p}^{b} (t) = \left[ {x_{N - p + 1}^{*} (t),x_{N - p}^{*} (t), \cdots ,x_{N - M - p + 2}^{*} (t)} \right]^{{\text{T}}}$, $p = 1,2, \cdots ,P$.

Step 2. According to formula (9) and (14), calculate the autocorrelation matrix ${\varvec{R}}_{p}^{f}$ and ${\varvec{R}}_{P - p + 1}^{b}$ ($p = 1,2, \cdots ,P$) for the forward and backward virtual subarrays, respectively.

Step 3. According to formula (16), the weighted virtual space smoothing matrix ${\varvec{R}}^{f4}$ is obtained.

## Experiments

In order to verify the performance of the proposed weighted space smoothing algorithm, the following simulation analysis is conducted. The simulation parameters are shown in Table 1.

**Table 1:** Simulation parameters.

Number of antenna elements	Element spacing	Number of snapshots	Carrier frequency (MHz)	Pulse repetition rate (Hz)	Target doppler shift (Hz)
16	$\lambda /2$	128	150	500	200

Antenna height	$R_{{\text{d}}}$(km)	Antenna horizontal 3 dB width (°)	Reflection coefficient	Angle search range	Angle search step
100	200	7.16	$0.9\exp ({\text{j}}\pi )$	− 10 ~ 10	0.1

Monte Carlo simulation times is ${\text{MC}} = 500$, and the number of the subarrays is $P = 5$. After decorrelation, the DOA estimation is obtained by use of the multiple signal classification (MUSIC) algorithm^21,22^.

If the difference between the incident angle of the interference signal and the desired signal is less than the beam width, it will cause an increase in angle estimation error, which is commonly known as the Rayleigh Resolution Limit. The MUSIC algorithm has the characteristic of exceeding the Rayleigh Resolution Limit and is a super-resolution DOA estimation method. However, the coherent signal sources have a significant impact on the performance of the MUSIC algorithm, mainly because they cause a decrease in the rank of the signal covariance matrix. The proposed weighted virtual space smoothing algorithm in this paper can effectively restore the rank of the signal covariance matrix, so that the rank of the signal covariance matrix is equal to the number of signal sources. Finally, combined with the MUSIC algorithm, the super-resolution DOA estimation results are obtained.

Define the angle estimate of the elevation angle of the direct wave as $\hat{\varphi }_{{\text{d}}}$, then the estimated RMSE is^23^

17 $${\text{RMSE}} = \sqrt {\frac{1}{{{\text{MC}}}}\sum\limits_{i = 1}^{{{\text{MC}}}} {(\hat{\varphi }_{{\text{d}}} (i) - \varphi_{{\text{d}}} )^{2} } }$$

After the estimated value $\hat{\varphi }_{{\text{d}}}$ is obtained, the height estimate $\hat{H}_{{\text{T}}}$ of the target can be expressed as

18 $$\hat{H}_{{\text{T}}} (i) = \sqrt {R_{{\text{d}}}^{2} + \left( {H_{{\text{A}}} + R_{{\text{e}}} } \right)^{2} - 2R_{{\text{d}}} \left( {H_{{\text{A}}} + R_{{\text{e}}} } \right)\cos \hat{\varphi }_{{\text{d}}} (i)} - R_{{\text{e}}}$$

where $$i = 1,2, \cdots ,{\text{MC}}$$.

The height measurement error is

19 $$\Delta H_{{\text{T}}} = \frac{1}{{{\text{MC}}}}\sum\limits_{i = 1}^{{{\text{MC}}}} {\left| {H_{{\text{T}}} - \hat{H}_{{\text{T}}} (i)} \right|}$$

**Figure 4:**
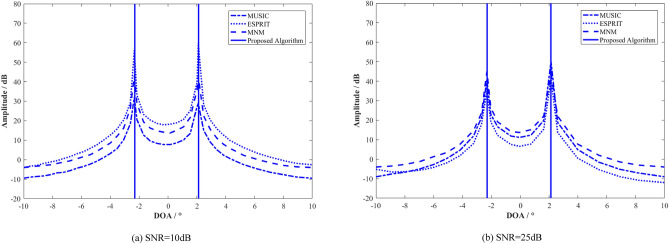
DOA angle estimation results of the improved FBSS algorithm proposed in this paper.

Figure 4 shows the angle estimation results of the MUSIC^24^, the estimated signal parameters via rotational invariance technique (ESPRIT)^25^, the mini-norm method (MNM)^26^ and the proposed algorithm in this paper. The real DOA angles in the simulation are $\varphi_{{\text{d}}} \approx 2.16^{ \circ }$ and $\varphi_{{\text{s}}} \approx - 2.26^{ \circ }$, respectively. Figure 4a shows the results when the signal to noise ratio (SNR) is 10 dB, and Fig. 4b shows the results when the SNR is 25 dB. In the figure, the two vertical lines are used to represent the angle value of multipath angle estimated by the proposed algorithm in this paper. It can be seen that the proposed algorithm in this paper can accurately estimate the angle of the coherent sources.

**Figure 5:**
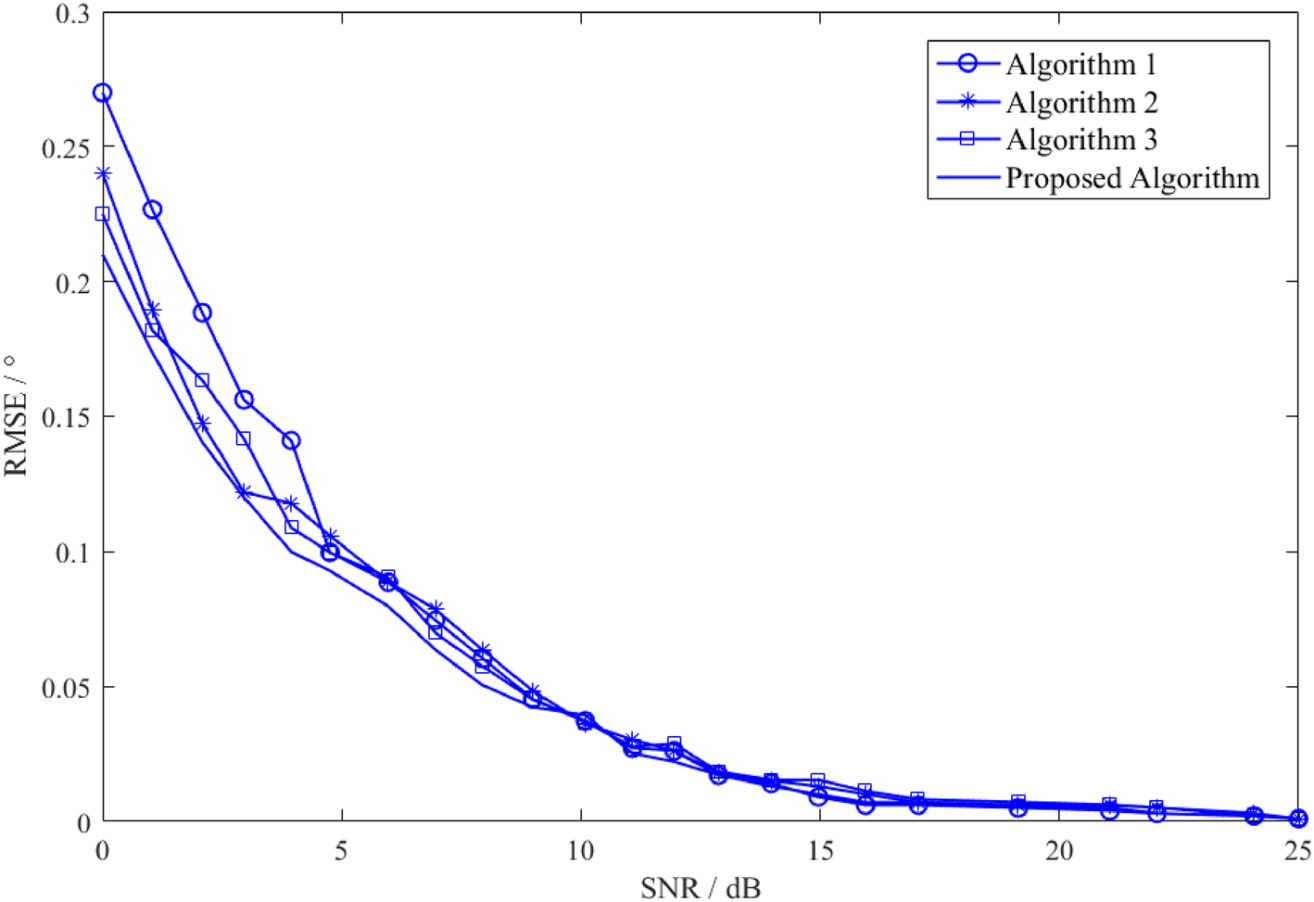
Results of angle estimation RMSE of various algorithms changing with SNR.

Figure 5 shows the change results of the angle estimation RMSE of the improved spatial smoothing algorithm and the other spatial smoothing algorithm with SNR. The algorithm 1, the algorithm 2 and the algorithm 3 represent the traditional FBSS algorithm, the improved spatial smoothing algorithm^27^, and the virtual space smoothing algorithm^28^, respectively. It can be seen that the RMSE of the traditional FBSS algorithm is the largest, and the algorithms of the algorithm 2 and the algorithm 3 are equivalent, but their performance is improved compared with the traditional FBSS algorithm, while the error of the improved FBSS algorithm in this paper is relatively minimum. It can also be seen that the angle measurement error of the traditional FBSS is the largest, and the angle measurement error of the improved FBSS algorithm in this paper is the smallest. Through the simulation analysis, it can be seen that the improved FBSS algorithm in this paper can obtain higher precision target height estimation when the multipath signals exist.

## Conclusion

The super resolution spatial spectrum estimation technology is an important technical means for the height measurement of meter wave phased array radar. However, due to the influence of multipath effect, the lobe splitting will occur during the height measurement of meter wave radar, which will increase the detection and measurement error of radar. In this paper, a height measurement method for meter wave radar based on the improved weighted spatial smoothing algorithm is proposed. This method uses the autocorrelation matrix of the output of the subarrays for cross correlation, and then uses the covariance matrix after the average of the forward and backward cross correlation matrices as the modified spatial smoothing matrix. The simulation results show that the proposed method can still achieve higher super resolution and smaller estimation error when the coherent sources are close to each other.

## Data Availability

The authors declare that all data generated or analysed during this study are included in this published article.

## References

[1] Narayanan VL, Hggstrm I, Mann I (2022). Effects of particle precipitation on the polar mesospheric summer echoes observed by EISCAT VHF 224MHz radar. Adv. Space Res..

[2] Kumar S, Rao TN, Radhakrishna B (2019). Identification and separation of turbulence echo from the multipeaked VHF radar spectra during precipitation. IEEE Trans. Geosci. Remote Sens..

[3] Liu YB, Wang CY, Gong J (2022). Robust suppression of deceptive jamming with VHF-FDA-MIMO radar under multipath effects. Remote Sens..

[4] Li CX, Chen BX, Yang ML (2017). Altitude measurement of low-elevation target for VHF radar based on weighted sparse Bayesian learning. IET Signal Process..

[5] Chen BX, Zhao GH, Zhang SH (2010). Altitude measurement based on beam split and frequency diversity in VHF radar. IEEE Trans. Aerosp. Electron. Syst..

[6] Guo R, Peng XY, Tian B (2022). Low elevation target altitude measurement for ubiquitous radar based on known transmitted waveform and sparse representation. IET Radar Sonar Navig..

[7] Chen GH, Chen BX (2013). Ambiguity resolution based on matrix completion in direction of arrival estimation for an interferometric array VHF radar. J. Electron. Inf. Technol..

[8] Li CX, Chen BX, Zheng YS (2017). Altitude measurement of low elevation target in complex terrain based on orthogonal matching pursuit. IET Radar Sonar Navig..

[9] Zhao GH, Shi GM (2011). Altitude measurement of low elevation target based on iterative subspace projection. IEEE Trans. Aerosp. Electron. Syst..

[10] Williams RT, Prasad S, Mahalanabis AK (1988). An improved spatial smoothing technique for bearing estimation in a multipath environment. IEEE Trans. Acoust. Speech Signal Process..

[11] Li J (1992). Improved angular resolution for spatial smoothing techniques. IEEE Trans. Signal Process..

[12] Wang BH, Wang YL, Chen H (2003). Weighted spatial smoothing algorithm for direction of arrival estimation of coherent sources. J. China Inst. Commun..

[13] Dai J, Ye Z (2011). Spatial smoothing for direction of arrival estimation of coherent signals in the presence of unknown mutual coupling. IET Signal Process..

[14] Pillai SU, Kwon BH (1989). Forward/Backward spatial smoothing techniques for coherent signal identification. IEEE Trans. Acoust. Speech Signal Process..

[15] Chen GH, Chen BX, Qin Y (2021). Robust height finding based on fractional lower order moments for an interferometric array very high frequency radar. J. Electron. Inf. Technol..

[16] Chen GH, Chen BX (2020). Robust altitude estimation based on spatial sign transform in the presence of diffuse multipath for very high frequency radar. J. Electron. Inf. Technol..

[17] Schelleng JC, Burrows CR, Ferrell EB (1933). Ultra-short wave propagation[J]. Bell Syst. Tech. J..

[18] Dong XD, Zhang XF, Zhao J (2021). DOA estimation for coprime array with mixed noise scenario via phased fractional low-order moment. IEEE Wirel. Commun. Lett..

[19] Wu, Y., Wang, C., Liu, J. Y. Underdetermined direction of arrival estimation with coprime array constrained by approximated zero norm. *Proc. of the IEEE International Conference on Signal Processing, Communications and Computing*, 1–5 (Macau, China, 2020)

[20] Cheng, B. L., Li, M. W., Feng, M. Y., et al. DOA estimation on CACIS-type array based on fast-Rvm algorithm. *Proc. of the International Conference on Systems and Informatics*, 1577–1582 (Shanghai, China, 2019).

[21] Wang GX, Zheng GM, Wang HZ (2022). Meter wave polarization-sensitive array radar for height measurement based on MUSIC algorithm. Sensors.

[22] Pang YC, Liu S, He Y (2021). A PE-MUSIC algorithm for sparse array in MIMO radar. Math. Prob. Eng..

[23] Wang XC, Jiang CS (2021). New calculation method for exact length weighting factor in cone-beam computed tomography. Int. J. Imag. Syst. Technol..

[24] Schmidt R (1986). Multiple emitter location and signal parameter estimation. IEEE Trans. Antennas Propag..

[25] Roy R, Kailath T (1989). ESPRIT-estimation of signal parameters via rotational invariance technique. IEEE Trans. ASSP.

[26] Turkmen, C., Secmen, M. Radar target classification for resonance scattering region targets with min-norm signal processing method. *Proc. of the IEEE Radar Methods and Systems Workshop*, 16–21 (Kyiv, USA, 2016).

[27] Dong M, Zhang SH, Wu XD (2008). An improved spatial smoothing technique. J. Electron. Inf. Technol..

[28] Liu ZG, Wang JK, Wang FL (2007). Virtual spatial smoothing algorithm. Acta Electron. Sin..

